# Spontaneous Tumor Lysis Syndrome in a Patient with a Dedifferentiated Endometrial Adenocarcinoma

**DOI:** 10.1155/2017/5103145

**Published:** 2017-08-27

**Authors:** Shinichi Harada, Keiki Nagaharu, Youichirou Baba, Tetsuya Murata, Toshiro Mizuno, Keiki Kawakami

**Affiliations:** ^1^Department of Hematology and Oncology, Suzuka General Hospital, Yamanohana 1275-53, Suzuka, Mie Prefecture 513-8630, Japan; ^2^Department of Hematology and Oncology, Mie University Hospital, Edobashi 2-174, Tsu, Mie Prefecture 514-0001, Japan; ^3^Department of Pathology, Suzuka General Hospital, Yamanohana 1275-53, Suzuka, Mie Prefecture 513-8630, Japan

## Abstract

Tumor lysis syndrome (TLS) is an oncological emergency caused by massive cytolysis of malignant cells. This syndrome eventually induces metabolic abnormalities. TLS is observed mainly among tumors with rapid cell proliferation or high sensitivity to antineoplastic treatment. In rare cases, TLS occurs without any cytotoxic treatment. Previous reports have shown that alternative stress including proceeding infection or an operation might play a role in TLS. However, exact mechanism of spontaneous TLS remains unknown. Here, we describe a case of a 59-year-old woman who presented with dedifferentiated endometrial adenocarcinoma and developed TLS without any cytotoxic chemotherapy. Although spontaneous TLS in solid malignancies are extremely rare, clinicians should consider the possibilities of TLS especially in aggressive solid tumors.

## 1. Background

Tumor lysis syndrome (TLS) is a life-threatening oncological emergency. Rapid cell death releases a large amount of nucleic acids, proteins, and electrolytes, leading to metabolic abnormalities such as hyperuricemia, hyperphosphatemia, hyperkalemia, and hypocalcemia [1]. This syndrome was first reported in 1929 [2] and has been commonly recognized as one of the comorbidities after chemotherapy. The key factors of TLS include a short doubling time, rapid proliferation, large tumor burden, disseminated tumor, and high sensitivity to antineoplastic drugs. However, it has been reported that some cases without therapeutic intervention would develop the TLS. Particularly, in hematological malignancies, there have been some cases of TLS. Spontaneous TLS cases in solid malignancy is extremely rare. Here, we present a rare case of dedifferentiated endometrial adenocarcinoma with spontaneous TLS.

## 2. Case Presentation

A 59-year-old woman was admitted to our hospital because of an 8-day history of fever and malaise. Her medical history included hypertension without medication. Upon admission, her vital parameters were blood pressure of 107/73 mmHg, pulse rate of 111 bpm, and body temperature of 37.0°C. She was alert (Glasgow coma scale; E4V5M6). She had a distended abdomen without tenderness. Pitting edema of the dorsum of the feet was seen. Other physical examinations were unremarkable. Laboratory tests (Table 1) showed an elevated white blood cell (WBC) count of 19300/*μ*L (normal range: 3200–9800/*μ*L), lactate dehydrogenase (LDH) activity of 444 IU/L (normal range: 100–250 IU/L), and C-reactive protein level of 14.48 mg/dL (normal range: 0.00–0.30 mg/dL). Human T-lymphotropic virus type 1 (HTLV-1) antibodies were positive without abnormal lymphocytes. Obtained blood and urinary culture revealed no pathogens.

**Figure 1:**
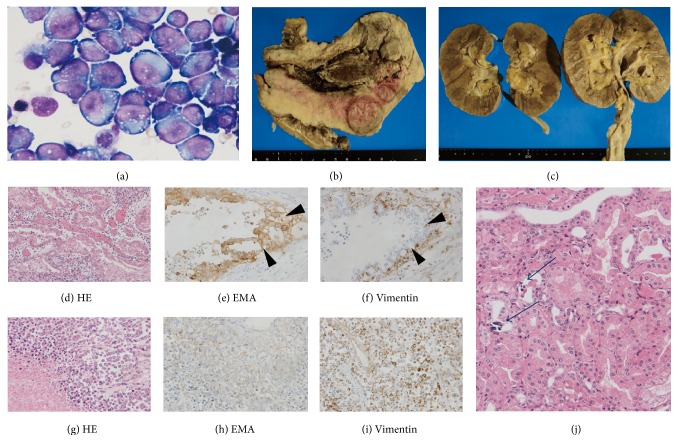
Histopathological findings of ascites and autopsy. (a) Cytomorphological evaluation of ascites revealed large abnormal cells mimicking lymphoma. (b, c) Autopsy showed an enlarged uterus. There was no apparent invasion in both kidneys. Small nodules were scattered throughout omentum. (d–i) Hematoxylin and eosin (HE) staining of the uterus showed two types of malignant cells (d, g) characterized by coexisting endometrial carcinoma and undifferentiated immature malignant cells. These endometrial malignant cells were positive for epithelial membrane antigen (EMA) (e) and negative for vimentin (f). Undifferentiated immature malignant cells were negative for EMA (h) and positive for vimentin (i). (j) HE staining of the kidney showed no malignant invasion and many small stones were seen in microtubules.

She was treated with hydration and ceftriaxone for the suspicion of urinary tract infection. Fever continued and urinary output declined gradually. Investigation by computed tomography revealed multiple masses in the uterus, ascites, disseminated small nodules, and right hydronephrosis because of the tumor. No atrophy was seen in either kidney. Cytomorphological evaluation of ascites revealed large atypical cells. These cells were scattered with loose adhesion mimicking malignant lymphoma (Figure 1(a)). Based on the cytological evaluation and positivity for HTLV-1 antibodies, she was suspected to have malignant lymphoma. Then, she was referred to hematology department.

On day 5 in hospital (the first day in the hematology department), her performance status was 3 (Eastern Cooperative Oncology Group). Her malaise and fever were sustained, and she developed anuria. Reevaluation of the laboratory tests showed elevated uric acid 18.8 mg/dL (normal range: <6.8 mg/dL), creatinine of 5.18 (normal range: <0.80 mg/dL), and LDH activity of 622 IU/L. Disseminated intravascular coagulopathy was also diagnosed (Table 1). We diagnosed the tumor lysis syndrome and she was treated with furosemide and rasburicase. Anuria did not improve despite these treatments, and continuous hemodialysis was induced.

On day 6 in hospital, she experienced a sudden cardiopulmonary arrest. Although cardiopulmonary resuscitation was performed, she expired. Autopsy indicated pulmonary embolism without intravascular invasion of tumor cells. Pathological evaluation for uterus showed differentiated endometrioid adenocarcinoma component with squamous differentiation (Figures 1(d), 1(e), and 1(f)) and undifferentiated carcinoma component (Figures 1(g), 1(h), and 1(i)). Epithelial membrane antigen (EMA) expression was diffusely positive in differentiated endometrioid adenocarcinoma lesion (Figure 1(e), arrowhead) but negative for undifferentiated carcinoma lesion (Figure 1(h)). On the other hand, vimentin staining was negative in differentiated adenocarcinoma lesion (Figure 1(f), arrowhead) and positive in undifferentiated carcinoma lesion (Figure 1(i)). Neuroendocrine tumor constitution was not observed. Diagnosis of the uterine tumor indicated dedifferentiated endometrial adenocarcinoma (DEAC). Disseminated malignant cells were also observed in her lungs, liver, greater omentum, and mesentery. These lesions showed invasion of round cells like those seen in ascites. The MIB-1 (proliferative) index was approximately 60%. Microcalcification was seen in proximal tubules to collecting ducts of bilateral kidneys (Figure 1(j)). Final diagnosis was spontaneous TLS caused by DEAC of the uterus.

## 3. Discussion

TLS usually refers to the constellation of metabolic disturbances that may follow the initiation of cancer treatment. The incidence of TLS has been reported as 4%–42% [28]. The variation of incidence depends on the types of malignancy. The TLS consensus panel recommended the risk-stratified prevention of TLS. In view of pathogenesis, the risk factors of TLS include tumor-related intrinsic and host-related extrinsic factors. Tumor-related intrinsic factors include a high proliferation rate, and host-related extrinsic factors are age, volume depletion, WBC count, and renal function [1]. Based on a PubMed search, at least 28 cases of spontaneous TLS have been reported in patients with solid tumors (Table 2). The literature search was using PubMed with the following medical keywords: {spontaneous} and {tumor lysis syndrome} OR {TLS}. These previous cases showed that several types of tumors have been reported to date. Contrary to the case of aggressive hematological tumors, not all cases have exhibited high proliferation. Although pathological findings of our case revealed high MIB-1 index, this value is not so high compared to that of hematological malignancies. These cases might have the host-related extrinsic factors. Some cases have been reported to be caused by host-related factors such as proceeding infection [29], the contrast dye iohexol [30], operations [31], and even anesthesia [32]. Our case has no apparent coexisting risks and we finally diagnosed her as having spontaneous TLS.

DEAC of the uterus is defined as containing both a low-grade endometrioid adenocarcinoma and undifferentiated carcinoma population. It has been reported as a rare but highly aggressive uterine cancer [33]. The undifferentiated component of DEAC sometimes can be confused with other tumors including lymphoma [34]. Taraif et al. reported that 80% of patients with DEAC die within 12 months of diagnosis [35]. Our case exhibited highly aggressive clinical course, as was suggested in several previous reports.

In conclusion, we experienced the extremely rare case of spontaneous TLS with DEAC. In the cases with DEAC, clinicians should pay attention to spontaneous TLS. Further investigations for new strategy to treat DEAC patients are needed.

## Figures and Tables

**Table 1:** Laboratory tests upon admission.

On admission		Fifth day		Reference range
Alb	3.3 g/dL	Alb	2.7 g/dL	3.5–4.5 g/dL
AST	16 IU/L	AST	35 IU/L	10–35 IU/L
ALT	7 IU/L	ALT	12 IU/L	10–35 IU/L
LDH	444 IU/L	LDH	662 IU/L	100–250 IU/L
BUN	17.8 mg/dL	BUN	66.4 mg/dL	<20 mg/dL
Cre	1.52 mg/dL	Cre	5.18 mg/dL	<0.80 mg/dL
Na	126 mEq/L	Na	126 mEq/L	135–145 mEq/L
K	4.9 mEq/L	K	5.7 mEq/L	3.5–4.5 mEq/L
Cl	89 mEq/L	Cl	83 mEq/L	95–108 mEq/L
UA	9.0 mg/dL	UA	18.8 mEq/L	<6.8 mEq/L
CRP	14.48 mg/dL	CRP	27.6 mg/dL	<0.30 mEq/L
		Ca	10.0 mg/dL	8.5–10.5 mg/dL
		P	9.3 mg/dL	2.7–4.6 mg/dL
CEA	5.7 ng/mL			0–5 ng/mL
CA19-9	2294 IU/mL			0–37 IU/mL
CA125	506 IU/mL			0–35 IU/mL
HTLV-1 antibody	Positive			Negative

**Table 2:** Review of spontaneous TLS in patients with solid tumors.

Tumor	Age	Sex	UA	K	Ca	P	Ref
			(mg/dL)	(mEq/L)	(mg/dL)	(mg/dL)	
Adenocarcinoma unknown origin	50	M	37	6.5	8.3	9.2	[3]
Adenocarcinoma unknown origin	59	F	26.5	ND	6.5	8.8	[4]
Adenocarcinoma unknown origin	71	F	10.3	5.78	9.6	6	[5]
Breast lobular carcinoma	62	F	10.1	ND	10.1	6	[6]
Cholangiocarcinoma	66	M	9.9	4.8	8.7	3.8	[7]
Colon cancer	82	F	20.4	ND	5.7	5.5	[8]
Gastric adenocarcinoma	36	M	16.9	5.6	7	6.9	[9]
Gastric adenocarcinoma	51	M	27.9	5.3	8.9	15.2	[10]
Germ cell tumor	13	F	28	5.6	7.2	7.3	[11]
Germ cell tumor	22	M	18	7.2	9.6	7.2	[12]
Germ cell tumor	52	M	21.8	7.9	5	7.1	[13]
Germ cell tumor	24	M	24	8.5	7.6	10	[13]
Hepatocellular carcinoma	72	M	20.1	4.5	7.2	5.4	[8]
Hepatocellular carcinoma	76	M	16.3	6.9	7.7	8.9	[14]
Hepatocellular carcinoma	70	M	22.9	6	11	6.9	[15]
Lung adenocarcinoma	72	M	12.6	7	8.2	8.3	[16]
Lung SCC	74	M	15.4	5.2	ND	4.7	[17]
Lung small cell lung cancer	53	M	8.3	6.1	ND	5.3	[18]
Maxillary SCC	53	M	20.9	7.6	6.2	11.8	[19]
Melanoma	69	M	24.6	6.3	8.4	3.8	[20]
Merkel cell cancer	87	F	13.9	5.6	7.3	7.2	[21]
Ovarian carcinoma	71	F	10.3	5.78	9.7	5.8	[22]
Pancreatic adenocarcinoma	56	F	14.3	7.5	4	6.3	[23]
Pheochromocytoma	80	M	16.5	6.6	8.4	5.8	[8]
Prostate cancer	72	M	28.1	4.9	8	8.3	[24]
Prostate cancer	56	M	16.4	5.7	10	11.7	[25]
Renal cell carcinoma	56	M	24.6	5	9.4	10.5	[26]
Sarcoma	49	F	14.3	5.1	7.7	6.9	[27]

## References

[1] Hochberg J., Cairo M. S. (2008). Tumor lysis syndrome: Current perspective. *Haematologica*.

[2] Bedrna J., Polcák J. (1929). Akuter harnleiterverschluss nach bestrahlung chronischer leukämien mit röntgenstrahlen. *Medizinische Klinik*.

[3] Crittenden D. R., Ackerman G. L. (1977). Hyperuricemic Acute Renal Failure in Disseminated Carcinoma. *Archives of Internal Medicine*.

[4] Saini N., Pyo Lee K., Jha S. (2012). Hyperuricemic Renal Failure in Nonhematologic Solid Tumors: A Case Report and Review of the Literature. *Case Reports in Medicine*.

[5] Wang Y., Yuan C., Liu X. (2014). Cutaneous metastatic adenocarcinoma complicated by spontaneous tumor lysis syndrome: A case report. *Oncology Letters*.

[6] Sklarin N. T., Markham M. (1995). Spontaneous recurrent tumor lysis syndrome in breast cancer. *American Journal of Clinical Oncology*.

[7] Mirrakhimov A. E., Ali A. M., Khan M., Barbaryan A. (2014). Tumor lysis syndrome in solid tumors: an up to date review of the literature. *Rare Tumors*.

[8] Vaisban E., Braester A., Mosenzon O., Kolin M., Horn Y. (2003). Spontaneous tumor lysis syndrome in solid tumors: really a rare condition?. *American Journal of the Medical Sciences*.

[9] Woo I. S., Kim J. S., Park M. J. (2001). Spontaneous acute tumor lysis syndrome with advanced gastric cancer. *Journal of Korean Medical Science*.

[10] Goyal H., Sawhney H., Bekara S., Singla U. (2014). Spontaneous Acute Tumour Lysis Syndrome in Gastric Adenocarcinoma: A Case Report and Literature Review. *Journal of Gastrointestinal Cancer*.

[11] Murray M. J., Metayer L. E., Mallucci C. L. (2011). Intra-abdominal metastasis of an intracranial germinoma via ventriculo-peritoneal shunt in a 13-year-old female. *British Journal of Neurosurgery*.

[12] D'Alessandro V., Greco A., Clemente C. (2010). Severe spontaneous acute tumor lysis syndrome and hypoglycemia in patient with germ cell tumor. *Tumori*.

[13] Pentheroudakis G., O'Neill V., Vasey P., Kaye S. (2001). Spontaneous acute tumour lysis syndrome in patients with metastatic germ cell tumours: Report of two cases. *Supportive Care in Cancer*.

[14] Kekre N., Djordjevic B., Touchie C. (2012). Cases: Spontaneous tumour lysis syndrome. *CMAJ*.

[15] Mehrzad R., Saito H., Krahn Z., Feinstein A. (2014). Spontaneous tumor lysis syndrome in a patient with metastatic hepatocellular carcinoma. *Medical Principles and Practice*.

[16] Feld J., Mehta H., Burkes R. L. (2000). Acute spontaneous tumor lysis syndrome in adenocarcinoma of the lung: A case report. *American Journal of Clinical Oncology: Cancer Clinical Trials*.

[17] Shenoy C. (2009). Acute spontaneous tumor lysis syndrome in a patient with squamous cell carcinoma of the lung. *QJM*.

[18] Kanchustambham V., Saladi S., Patolia S., Stoeckel D. (2017; 9). Spontaneous tumor lysis syndrome in small cell lung cancer. *Cureus*.

[19] Abboud M., Shamseddine A. (2009). Maxillary Sinus Squamous Cell Carcinoma Presenting with Fatal Tumor Lysis Syndrome: A Case Report and Review of the Literature. *Case Reports in Oncology*.

[20] Mouallem M., Zemer-Wassercug N., Kugler E., Sahar N., Shapira-Frommer R., Schiby G. (2013). Tumor lysis syndrome and malignant melanoma. *Medical Oncology*.

[21] Grenader T., Shavit L. (2011). Tumor lysis syndrome in a patient with merkel cell carcinoma and provoked pathologic sequence of acute kidney injury, reduced clearance of carboplatin and fatal pancytopenia. *Onkologie*.

[22] Okamoto K., Kinoshita T., Shimizu M. (2015). A case of spontaneous tumor lysis syndrome in a patient with ovarian cancer. *Case Reports in Obstetrics and Gynecology*.

[23] Saleh R. R., Rodrigues J., Lee T. C. (2015). A tumour lysis syndrome in a chemotherapy naïve patient with metastatic pancreatic adenocarcinoma. *BMJ Case Reports*.

[24] Lin C.-J., Hsieh R.-K., Lim K.-H., Chen H.-H., Cheng Y.-C., Wu C.-J. (2007). Fatal spontaneous tumor lysis syndrome in a patient with metastatic, androgen-independent prostate cancer. *Southern Medical Journal*.

[25] Serling-Boyd N., Quandt Z., Allaudeen N. (2017). Spontaneous tumor lysis syndrome in a patient with metastatic prostate cancer. *Molecular and Clinical Oncology*.

[26] Norberg S. M., Oros M., Birkenbach M., Bilusic M. (2014). Spontaneous tumor lysis syndrome in renal cell carcinoma: a case report. *Clinical Genitourinary Cancer*.

[27] Zakharia Y., Mansour J., Vasireddi S. (2014). Tumor Lysis Syndrome in a Retroperitoneal Sarcoma. *Journal of Investigative Medicine High Impact Case Reports*.

[28] Coiffier B., Altman A., Pui C.-H., Younes A., Cairo M. S. (2008). Guidelines for the management of pediatric and adult tumor lysis syndrome: An evidence-based review. *Journal of Clinical Oncology*.

[29] Chen R.-L., Chuang S.-S. (2009). Transient spontaneous remission after tumor lysis syndrome triggered by a severe pulmonary infection in an adolescent boy with acute lymphoblastic leukemia. *Journal of Pediatric Hematology/Oncology*.

[30] Yun S., Vincelette N. D., Phan T., Anwer F. (2014). Spontaneous tumour lysis syndrome associated with contrast dye iohexol use in mantle cell lymphoma. *BMJ Case Reports*.

[31] Verma A., Mathur R., Chauhan M., Ranjan P. (2011). Tumor lysis syndrome developing intraoperatively. *Journal of Anaesthesiology Clinical Pharmacology*.

[32] Farley-Hills E., Byrne A. J., Brennan L., Sartori P. (2001). Tumour lysis syndrome during anaesthesia. *Paediatric Anaesthesia*.

[33] Silva E. G., Deavers M. T., Bodurka D. C., Malpica A. (2006). Association of low-grade endometrioid carcinoma of the uterus and ovary with undifferentiated carcinoma: A new type of dedifferentiated carcinoma?. *International Journal of Gynecological Pathology*.

[34] Park S. Y., Park M. H., Ko H. S. (2014). Dedifferentiated endometrioid adenocarcinoma of the uterus: Highly aggressive and poor prognostic tumor. *Korean Journal of Pathology*.

[35] Taraif S. H., Deavers M. T., Malpica A., Silva E. G. (2009). The significance of neuroendocrine expression in undifferentiated carcinoma of the endometrium. *International Journal of Gynecological Pathology*.

